# Enhanced Antibacterial Activity of Poly (dimethylsiloxane) Membranes by Incorporating SiO_2_ Microspheres Generated Silver Nanoparticles

**DOI:** 10.3390/nano9050705

**Published:** 2019-05-06

**Authors:** Qihui Shen, Yixuan Shan, Yang Lü, Peng Xue, Yan Liu, Xiaoyang Liu

**Affiliations:** 1 Department of Chemistry and Pharmaceutical Engineering, Jilin Institute of Chemical Technology, Jilin 132022, China; shenqhui@gmail.com (Q.S.); syx13654354458@hotmail.com (Y.S.); lvyang198511@gmail.com (Y.L.); 2 State Key Laboratory of Inorganic Synthesis and Preparative Chemistry, College of Chemistry, Jilin University, Changchun 130012, China; xuepeng16@mails.jlu.edu.cn

**Keywords:** silver nanoparticles, silica dioxide microspheres, PDMS membranes, antibacterial activity

## Abstract

The nonspecific adsorption of proteins and bacteria on the surface of polydimethylsiloxane (PDMS) had been a serious concern in a wide range of applications, such as medical devices. In order to improve the anti-adhesive and antibacterial capability, bare silver nanoparticles (AgNPs, ~15 nm) were generated in-situ on their surface without extra reducing and stabilizing agents. The main reason for this was that the SiO_2_ microspheres that are covalent bonded to the bulked PDMS could not only generate AgNPs spontaneously but also insure that no AgNPs were released to the environment. Meanwhile, the thiol-group-functionalized SiO_2_ microspheres self-assembled on the surface of PDMS by thiol-vinyl click reaction without any impact on their biomedical applications. After the modification of SiO_2_ microspheres with AgNPs, the surface of PDMS showed a smaller water contact angle than before, and the adhesion and growth of *E. coli* and *Bacillus subtilis* were effectively inhibited. When the monolayer of SiO_2_ microspheres with AgNPs was assembled completely on the surface of PDMS, they present improved bacterial resistance performance (living bacteria, 0%). This approach offers an antibacterial and anti-adhesive surface bearing small and well-defined quantities of in-situ generated AgNPs, and it is a novel, green, simple, and low-cost technique to generate AgNPs on soft biomedical substrates.

## 1. Introduction

Polydimethylsiloxane (PDMS) is optically clear, inert, non-toxic, and one of the most widely used silicon-based organic polymers, which have been applied in contact lenses [1], elastomers [2], cosmetics [3], food [4], medicine [5], and medical devices [6]. However, although they have excellent stability and biocompatibility for practical applications, the silicones usually have nonspecific surface adsorption of proteins and bacteria, which could be a big disadvantage in many applications such as medical devices [7]. The nonspecific microbial adhesion to medical devices made from silicones may cause formation of biofilms on the material surface and the subsequent infections [8]. Recently, many strategies have been employed to improve the antifouling performance of PDMS, including surface modification with biomimetic components coating [9,10]. However, these modified hybrids often suffered from limitations, such as weak interfacial bonding and low robustness [11,12]. These materials are incapable of acting against bacteria once they adhere onto the surface, so an ideal antifouling material should present both anti-adhesive and antibacterial capability.

Known for the outstanding antibacterial effects against a broad spectrum of bacteria, nanomaterials have been widely used to functionalize a range of materials and surfaces [13,14], especially silver nanoparticles (AgNPs) [15,16]. Although the antibacterial mechanisms of AgNPs remain controversial, it is generally accepted that the silver works by damaging bacterial cell membranes and interfering with basic metabolic functions of microbes [17]. Another advantage of using AgNPs as antibacterial agent is that the bacterial resistance to silver is rare and develops slowly in comparison to the resistance to antibiotics [18].

Antibacterial surfaces based on AgNPs could be achieved by a wide variety of methods, while two of the most popularly used were physical deposition [19,20] and chemical reduction [21,22]. More importantly, in most of above methods, AgNPs were weakly bound to the surface of PDMS with NPs easily released during the application, for example, in body fluids or human tissues [23,24,25]. For their practical application, the ideal AgNPs-based antibacterial biomaterials should cause minimal damage to healthy human cells, have AgNPs tightly bound to the material surface, and have the capability to control the AgNPs density on the surface of biomaterials. More importantly, they should release only silver and not organic ligands if bacteria are present, in order to maximize the antibacterial effect from AgNPs.

To overcome the challenge of loading the AgNPs antibacterial and anti-adhesive surface to PDMS, we designed the silica microspheres (SMs) with in-situ growth of AgNPs on their surface, and the size of the as-prepared AgNPs (~15 nm) could be controlled by tuning the concentration of Ag^+^ ions in our recent report [26]. In this paper, these SMs were covalently bonded to the bulk PDMS and bare AgNPs would be spontaneously generated on the surface of the prepared SMs through the reduction process of Ag^+^ by thiol groups. This approach produced antibacterial materials with anti-adhesive surfaces coated with small and well-defined quantities of in-situ generated AgNPs, and is a novel, green, simple, and low-cost technique to generate AgNPs on the soft biomedical substrates.

We chose click chemistry to functionalize the surface of PDMS [27,28], which was simple, mild and bio-friendly. Upon UV or visible light irradiation, the thiol groups on the surface of SMs, as one clickable function group, would anchor the SMs to the surface of PDMS by expediently combining with the other clickable function groups (e.g., terminal vinyl groups) modified on the surface of PDMS membranes.

## 2. Materials and Methods

### 2.1. Materials

3-Mercaptopropyl trimethoxysilane (MPTMS, 97%) was obtained from J&K Scientific Ltd. (Beijing, China). Allyltriethoxysilane (ATOS) was purchased from TCI Development Co. Ltd. (Shanghai, China). Silver nitrate (AgNO_3_, 99.8%), ammonium hydroxide (NH_4_OH, 28%), sulphuric acid (H_2_SO_4_, 98%), hydrogen peroxide (H_2_O_2_, 30%) were obtained from Sinopharm Chemical Reagent Co. Ltd. (Shanghai, China) Tryptic soy broth medium (TSB) and tryptic soy agar medium (TSA) were purchased from Hangzhou Microbial Reagent Co., Ltd. (Hangzhou, China) Fluorescent dyes Hoechst33342 and PI were purchased from Beijing Solarbio science & technology Co., Ltd.(Beijing, China) All the reagents were used without further purification.

### 2.2. Synthesis of AgNPs on the Surface of PDMS which Assembled with SiO_2_ Microspheres

#### 2.2.1. Surface of PDMS Modified with Ene Group

PDMS slides (thickness 0.8 mm, prepared by the authors) were cleaned with piranha solution for 5 min and then washed for four times with purified water under ultrasonic. Subsequently, the PDMS were washed with ethanol and immersed into 50 mL ATOS (1 mmol/L) solution for 20 min, then, the PDMS were washed twice with ethanol.

#### 2.2.2. Synthesis of SMs Functionalized with Thiol Groups

Thiol-terminated SMs (~1 μm) were synthesized by remodeled sol-gel process as a previously published method [26]. Briefly, 0.1 g MPTMS was mixed with 10 g water under vigorous stirring until MPTMS droplets completely disappeared, and then NH_4_OH was added to the mixture solution to adjust the pH to 11. The reaction continued for 48 h at room temperature. The precipitates were centrifugal washed twice with ethanol and re-dispersed in water.

#### 2.2.3. Assemblies of SMs on the Surface of PDMS (PDMS-SMs) by Light Click Chemistry Reaction

The SMs (1 mg) were placed in a 6” diameter Petri dish and dispersed into 20 mL water, and PDMS slides were immersed in this suspension. The dish was placed in a larger container with water at its bottom and the top of the container was covered with a clear plastic wrap. Then the samples were gently shaken on a reciprocating platform shaker and exposed to 365 nm UV light for at least 30 min. After the reaction, the PDMS slides were rinsed with copious amounts of purified water, dried with a stream of nitrogen.

#### 2.2.4. Synthesis of AgNPs on the Surface of PDMS-SMs (PDMS-SMs-AgNPs)

The PDMS-SMs was immersed in 20 mL of AgNO_3_ (0.1 mol/L) solution in the dark. Within 5 min, the surface color of PDMS-SMs changed from transparent to yellow. Then the slides were cleaned with water and dried in air or kept in ethanol for future application.

### 2.3. Characterization of Samples

Transmission electron microscopy (TEM) and High resolution transmission electron microscope (HRTEM) characterizations were conducted by using a Tecnai G2 S-Twin F20TEM (FEI, Hillsboro, OR, USA), operating at 200 KV. The SMs with AgNPs were scraped from PDMS and suspended in ethanol and supported onto a holey carbon film supported with Cu grid, then were dried in air for TEM measurement. Atomic force microscopy (AFM) images were obtained by the Bruker’s dimension FastScan atomic force microscope (Bruker, Santa Barbara, CA, USA) in air. X-ray photoelectron spectroscopy spectra (XPS) were recorded on a AXIS UltraDLD (Kratos Analytical Ltd., Manchester, UK) ultrahigh vacuum surface analysis system (aluminum Kα source: 1486.6 eV) at room temperature. Fourier Transform infrared spectra (FT-IR) were recorded by using Spectrum One FT-IR spectrometer (PerkinElmer Inc., Boston, MA, USA) and KBr was used as reference. Water contact angles (WCA) were measured using a JC2000D drop shape analysis instrument (Zhongchen Digital Technic Apparatus Co, Ltd, Shanghai, China) under ambient humidity and temperature. Microscope images were measured using DM4000B fluorescent upright microscope (,Leica, Buffalo Grove, IL, USA) which equipped with a digital color camera (Leica CDA), a broad-band ultraviolet (330–385 nm) light source (100 W mercury lamp), and a long-pass interference filter (DM 400, Chroma Tech, Brattleboro, VT, USA). The material surface performance was carried out in a CFT-1 tribometer (Zhongke Kaihua Technology Development Co. Ltd., Lanzhou, China) in a dry environment under a 20-N load (1085 high carbon steel ball with diameter of 4 mm as friction pair, and one-way displacement 5 mm were also conducted at fixed speed (500 r/min) within 40 min).

### 2.4. Study of Antibacterial Activity

*Escherichia coli* (*E. coli*) and *Bacillus subtilis* were Gram-negative and Gram-positive bacteria respectively and bacteria were incubated in a TSB liquid culture medium and shake at 37 °C for 12–16 h. After sterilization, the test was initiated by pouring the TSA onto sterilized Petri dishes and was allowed to solidify, we adjusted the concentration of the bacterial suspension until the OD_600_ ~ 0.1. Added the bacterial suspension 10 μL inoculated onto the entire surface of an agar. Each film sample was placed on the TSA surface [29]. The Petri dishes were incubated at 37 °C for 24 h and the clear zone were measured to determine the antibacterial activity. The antibacterial studies were repeated 3 times.

Bacterial was treated with fluorescent dyes Hoechst33342 and PI to visualize the adhesion and survival rate of *E. coli* and *Bacillus subtilis*. Briefly, 1 mL of cell buffer solution, 5 μL of Hoechst33342, and 5 μL PI staining reagent mixture were added to each sample. Finally, the strips were stained with backlight bacterial staining and ice-water bath for 30 min, washed with sterile PBS (phosphoric acid buffer) [30] for several times, and tested by the fluorescent upright microscope. The images were recorded by a CCD camera on a Leica DM4000B.

## 3. Results and Discussion

**Figure 1:**
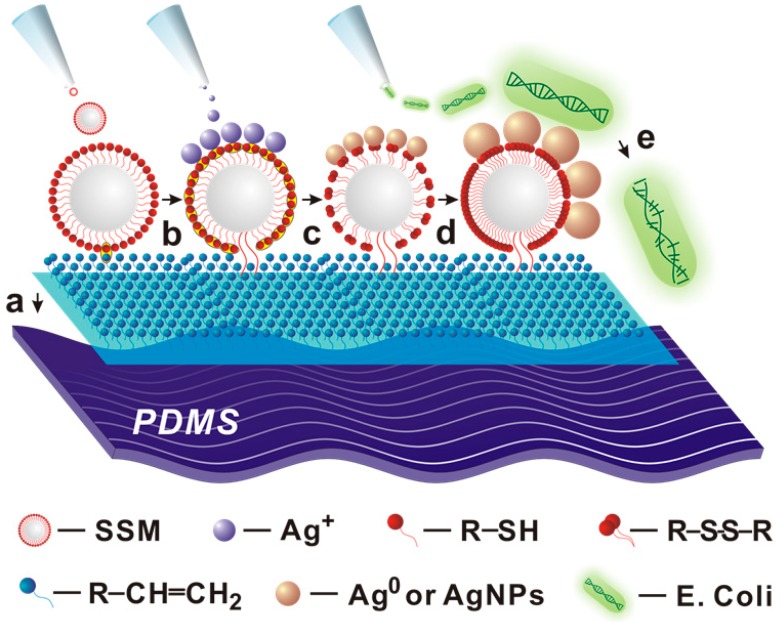
Schematic illustration of procedures for preparation of polydimethylsiloxane (PDMS)-supported silver nanoparticles (AgNPs) against *E. coli*. **a**) silica microspheres (SMs) were generated by the hydrolysis of 3-Mercaptopropyl trimethoxysilane (MPTMS); **b**) the SMs were assembled on the surface of PDMS by thiol-vinyl click reaction; c) the silver ions were reduced by the thiols groups; d) more and more silver atoms assembled together into AgNPs; e) PDMS-supported AgNPs had higher antibacterial activity against *E. coil*.

As shown in Figure 1, the bulk PDMS were firstly modified homogeneously to form a layer of terminal vinyl groups on their surface. Since each silane coupling chain had one vinyl group, which offered high immobilization density for SMs. When the SMs dropped on the vinyl groups modified PDMS, the thiol-vinyl click reaction took place with the formed thioether bonds and then SMs were immobilized on the surface of PDMS by covalent bonds, which could efficiently avoid the escape of SMs from the surface of PDMS. It should be noted that the embedded SMs could absorb Ag^+^ ions, and reduced Ag^+^ ions into Ag^0^ with the thiol groups [26]. Within one minute, the nucleation and growth of AgNPs can spontaneously complete without other reducing or capping agents. Benefitting from the exposition on the surface of PDMS-SMs-AgNPs, the bare and ultrafine AgNPs showed excellent antibacterial performances against both Gram-negative/positive bacteria.

### 3.1. Characterization

**Figure 2:**
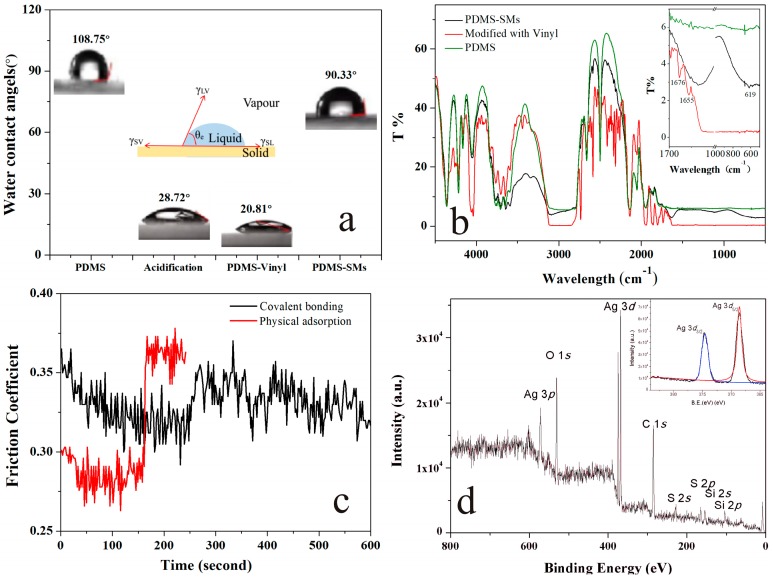
(**a**) Water contact angle (WCA) measurement of the PDMS and functionalized PDMS strip. (**b**) Fourier Transform infrared (FTIR) spectra of the PDMS (green line), PDMS-vinyl (red line) and PDMS-SMs (black line). (**c**) Effects of SMs modified PDMS on friction coefficient. The SMs were assembled on the surface of PDMS by covalent bonds (black line) or physical adsorption (red line); (**d**) X-ray photoelectron spectroscopy (XPS) survey spectrum of the SMs-AgNPs (insert: Ag 3d spectrum).

When the surface of PDMS was modified with vinyl groups, their surface properties changed significantly, as shown in Figure 2a. A material surface with a water contact angle (WCA) above 90° was deemed to be hydrophobic, while that below 90° was deemed to be hydrophilic [31]. After the surface of PDMS was pre-treated with sulfuric acid, the WCA decreased from 108.75° to 28.72° and it presented high hydrophilicity because so many hydroxyl groups had been created. When the acidified PDMS were dipped in the solution of silane coupling agent with vinyl groups, the vinyl groups could be bonded on the surface of PDMS and the WCA further decreased to 20.81°. However, when the SMs assembled and covalently bonded onto the vinyl groups on the PDMS surface via click reaction under UV irradiation, the WCA increased to 90.33° and the surface of PDMS became hydrophobic again. Meanwhile, the SMs modified PDMS were ready to provide enough thiol groups for growth of the AgNPs. 

The FT-IR spectrum also confirmed this mechanism, as shown in Figure 2b. Firstly, the peak of ‒OH at 3691 cm^−1^, ‒OH at 3644 cm^−1^, demonstrated that the surface of PDMS was active to functionalized modified by the hydrolyze of silanes [32]. Secondly, the cross-linking of vinyl groups onto the PDMS surface was confirmed via the characteristic absorption peaks at 1676 cm^−1^ and 1655 cm^−1^, which were assigned to the ‒CH=CH_2_ group [33]. Finally, the peak at 619 cm^−1^ was attributed to the C–S stretch [34] and proved that the SMs coated PDMS were covalently bonded.

The method for controlling the self-assembly of a compact monolayer of SMs at the surface of PDMS was the key to enhancing their antibacterial activity. Self-assembly of SMs relied on the spontaneous diffusion and localization of SMs to the PDMS surface as well as an increased efficiency of bonding between thiol (on SMs) and vinyl groups (on PDMS) via the click reaction. When the PDMS strip was made into square of 1 cm in side length and the thickness was 0.8 mm, the aspect ratio of the SMs was at a concentration of 4.35 ± 0.02 × 10^7^ particles/mL (the concentration of SMs: surface area of PDMS strip = 2.63 ± 0.02 × 10^7^ particles/cm^2^). As shown in Figure S1a–d, when the concentration of SMs was below 4.35 ± 0.02 × 10^7^ particles/mL, the sub-monolayer coverage was obtained, while higher concentration would induce aggregation of SMs (Figure S1f) on the surface of PDMS. In Figure S1e, a full monolayer surface coverage of the SMs could be obtained at the concentration of 4.35 ± 0.02 × 10^7^ particles/mL.

To test the stability of SMs bonded to the PDMS, the strips of PDMS-SMs (covalent bonding, CB) and SMs dropped on the surface of PDMS (physical adsorption, PA) were prepared. The friction coefficient of the PDMS coated SMs were carried out in the CFT-1 tribometer (Figure 2c) [35]. In the first 160 s, the friction coefficient of CB ranged from 0.302 to 0.365, while the friction coefficient of PA was between 0.291–0.301. After 160 s, the friction coefficient of PA increased significantly because of the SMs shedding from the surface of PDMS and the grated PDMS, while the friction coefficient of CB did not fluctuate greatly during the 10 min due to the stable covalent bonding between SMs and PDMS. Therefore, this method can efficiently avoid the leakage of AgNPs and expand the applications of PDMS in medical devices.

**Figure 3:**
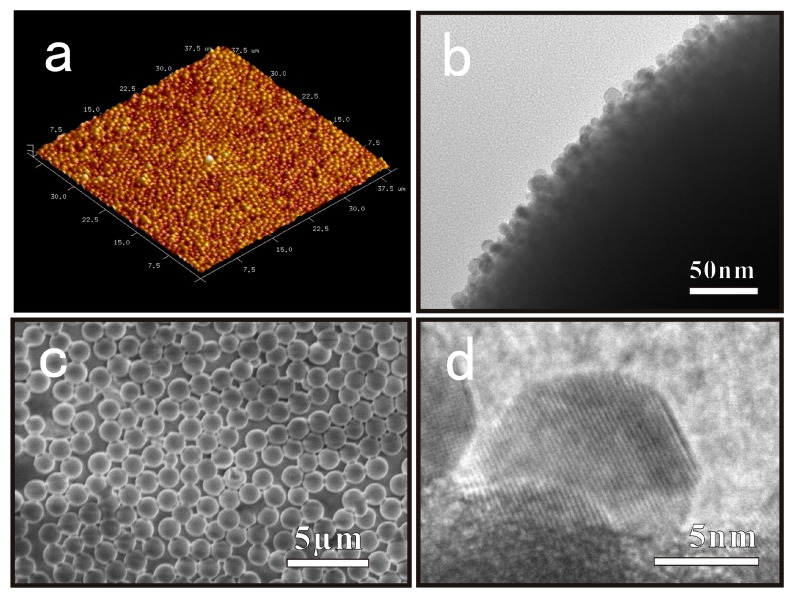
Images of PDMS-SMs-AgNPs. (**a**) The AFM image of SMs with AgNPs formed on the surface of PDMS; (**b**) The HRTEM images of AgNPs supported on SMs; (**c**) SEM images of SMs with AgNPs loading on the surface of PDMS; (**d**) The interplanar spacing of the lattice fringes of AgNPs.

As soon as the solution containing Ag^+^ ions dropped onto the surface of the SMs coated PDMS, the in-situ nucleation and growth of AgNPs were completed on the surface of SMs spontaneously. Without other reducing agents or capping agents, thiol groups of SMs could reduce the Ag^0^ formation energy, transport electrons efficiently, improve the nucleation density, and protect AgNPs against oxidation [36,37,38]. Figure 3c,d shows the AgNPs generated on the surface of the SMs, and their interplanar spacing of the lattice fringe was 0.236 nm, consistent with that of the (111) facet of metallic silver with face-centered-cubic structure [39]. Meanwhile, the XPS of the sample showed peaks at 374.1 and 368.1 eV, which could be assigned to the Ag 3d_3/2_ and 3d_5/2_ of metallic silver respectively as shown in Figure 2d [40,41].

### 3.2. Antimicrobial Efficiency

**Figure 4:**
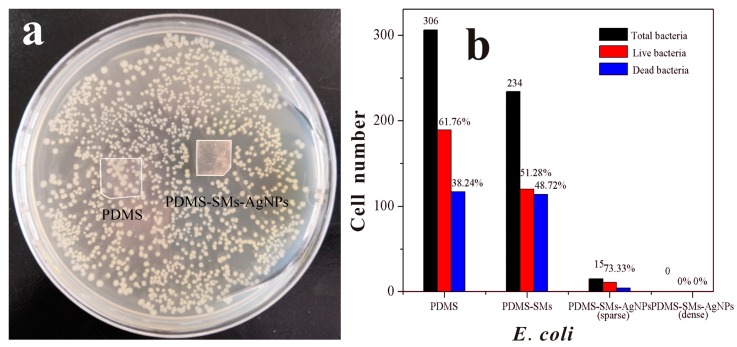
ZOI assay of PDMS and PDMS-SMs-AgNPs by *E.coli* after 24 h incubation at 37 °C (**a**). Cell numbers of *E. coli* growing on the PDMS, PDMS-SMs, sparse/tight SMs-AgNPs modified PDMS (**b**). There was no *E. coli* observed on the tight PDMS-SMs-AgNPs.

Antimicrobial efficiency of the silver nanoparticles coated PDMS films against bacterial was carried out by zone of inhibition (ZOI) assay, which were selected as the indicators for antibacterial evaluation. After the incubation of *E. coli* (Gram−) and *Bacillus subtilis* (Gram+) on the surface of the PDMS-SMs-AgNPs and PDMS in agar medium for 24 h, the clear zone (1.87 ± 0.05 mm) formed around the samples was recorded as an indication of inhibition of the microbial species in Figure 4a and Figure S2 respectively. The ZOI of the PDMS-SMs-AgNPs indicates the antimicrobial efficiency of these films with less silver loading (about 14.5 μg/cm^2^) compared with the other silver antibacterial materials as shown in Table 1, while there was no clear zone for the control sample (PDMS) as shown in Figure 4a. Therefore, this in *vitro* result showed that the presence of PDMS-SMs-AgNPs strip had excellent antibacterial effects against Gram+/Gram− bacteria.

To test the adhesive level of bacteria to the material surfaces, PDMS-SMs-AgNPs and PDMS were respectively cultured with bacteria after 24 h incubation at 37 °C, and fluorescent dyes were applied to stain bacteria. The dead bacteria were stained by PI staining (red), while the Hoechst 33342 (blue) helped to identify all bacteria. As shown in Figure S2a–d, the blue fluorescent channel presented all bacteria that were adsorbed on the material surfaces, while the red channel indicated dead bacteria. On PDMS, a decent amount of blue fluorescence was observed, and after counting the number of the live bacteria vs. total bacteria with the optical microscope, live *E. coli* still took 61.76% of the total bacterial that were adsorbed on the surface. The SMs functionalized PDMS showed an even larger area of blue fluorescence, indicating the high level adsorption of bacteria on the surface; with live *E.coli*, it removed 51.28% of the total bacteria. On the other hand, PDMS-SMs-AgNPs sample showed little to no *E.coli* adhesion whether the SMs-AgNPs sparsely (< 15) or densely covered the PDMS surface as shown in Figure 4b. Besides, the numbers of *Bacillus subtilis* adsorbed to PDMS, PDMS-SMs, PDMS-SMs-AgNPs were consistent with *E. coli* in Figure S3–S5.

## 4. Conclusions

In this work, we successfully applied the SMs-AgNPs to modify the PDMS surface via click reaction and showed that the PDMS-SMs-AgNPs surface prohibited the adhesion and growth of *E. coli* and *Bacillus subtilis* effectively. The surface of PDMS-SMs-AgNPs showed hydrophobicity. Meanwhile, SMs assembled on the surface of PDMS by the thiol-vinyl click reaction and AgNPs in-situ generated on the surface of SMs assemble PDMS could restrain the bacteria growth and even the leakage of AgNPs. However, further work is still needed to promote the future use of SMs on the surface of PDMS as a new generation of powerful antibacterial agents with wide-ranging applications.

## Figures and Tables

**Table 1:** Zone of inhibition (ZOI) of silver nanomaterials against *E. coli* and *B. subtilis.*

Agent	Silver Dosage (μg/cm^2^)	ZOI (diameter, cm)		ZOI/Dosage (cm^3^/μg)		Ref.
		*E. coli*	*B. subtilis*	*E. c*oli	*B. subtilis*	
Ag/ZnO	10455	2.3 ± 0.06	2.5 ± 0.12	2.2 × 10^−4^	2.4 × 10^−4^	[1]
AgNPs/ Coffea extract	1892	3.1	2.7	1.6 × 10^−3^	1.4 × 10^−3^	[2]
Silver Catheter	-	No ZOI	0.73 ± 0.21	-	-	[3]
Dopa-PAA/PEI-0.05AgNPs	128	-	0.26 ± 0.04	-	1.8 × 10^−3^	[5]
AgNPs/ PCL/PDMAEMA	126	1.02	-	8.2×10^−3^	-	[6]
AgNPs@oCNT-Nanohybrid	77	0.51	-	6.6 × 10^−3^	-	[42]
PDMS- SMs-AgNPs	14.5	0.98 ± 0.02	1.16 ± 0.04	6.7 × 10^−2^	8.0 × 10^−2^	This work

## Supplementary Materials

The following are available online at https://www.mdpi.com/2079-4991/9/5/705/s1, Figure S1: Optical microscope graph of PDMS with different concentrations of SMs-AgNPs were assembled on their surface. Figure S2: Growth of *E. coli* on the surface of PDMS, PDMS-SMs, PDMS-SMs-AgNPs (sparse/tight). Figure S3: ZOI assay of PDMS and PDMS-SMs-AgNPs with *Bacillus subtilis* after 24 h incubation at 37  °C. Figure S4: Growth of *Bacillus subtilis* on the surface of PDMS, PDMS-SMs, PDMS-SMs-AgNPs. Figure S5: Cell numbers of *Bacillus subtilis* growing on the PDMS, PDMS-SMs, sparse/tight SMs-AgNPs modified PDMS.
