# Supplementary material for: Enhanced Antibacterial Activity of Poly (dimethylsiloxane) Membranes by Incorporating SiO2 Microspheres Generated Silver Nanoparticles
Source: Nanomaterials (Basel). 2019 May 6;9(5):705. doi: 10.3390/nano9050705 (PMC6566769; doi:10.3390/nano9050705)
Supplement: Supplementary file 1 [file nanomaterials-09-00705-s001.pdf]

Enhanced Antibacterial Activity of poly (dimethylsiloxane) Membranes by Incorporating SiO_2 Microspheres Generated Silver Nanoparticles

Qihui Shen [1,2], Yixuan Shan [1], Yang Lü [1], Peng Xue [2], Yan Liu [1,2,*] and Xiaoyang Liu [2,*]

[1] Department of Chemistry and Pharmaceutical Engineering, Jilin Institute of Chemical Technology, Jilin 132022, China; shenqhui@gmail.com (Q.S.); syx13654354458@hotmail.com (Y.S.); lvyang198511@gmail.com (Y.L.)

[2] State Key Laboratory of Inorganic Synthesis and Preparative Chemistry, College of Chemistry, Jilin University, Changchun 130012, China; xuepeng16@mails.jlu.edu.cn

* Correspondence: ly@jlict.edu.cn (Y.L.); liuxy@jlu.edu.cn (X.L.); Tel.: +86-432-62185233 (Y.L.); +86-431-85168316 (X.L.)

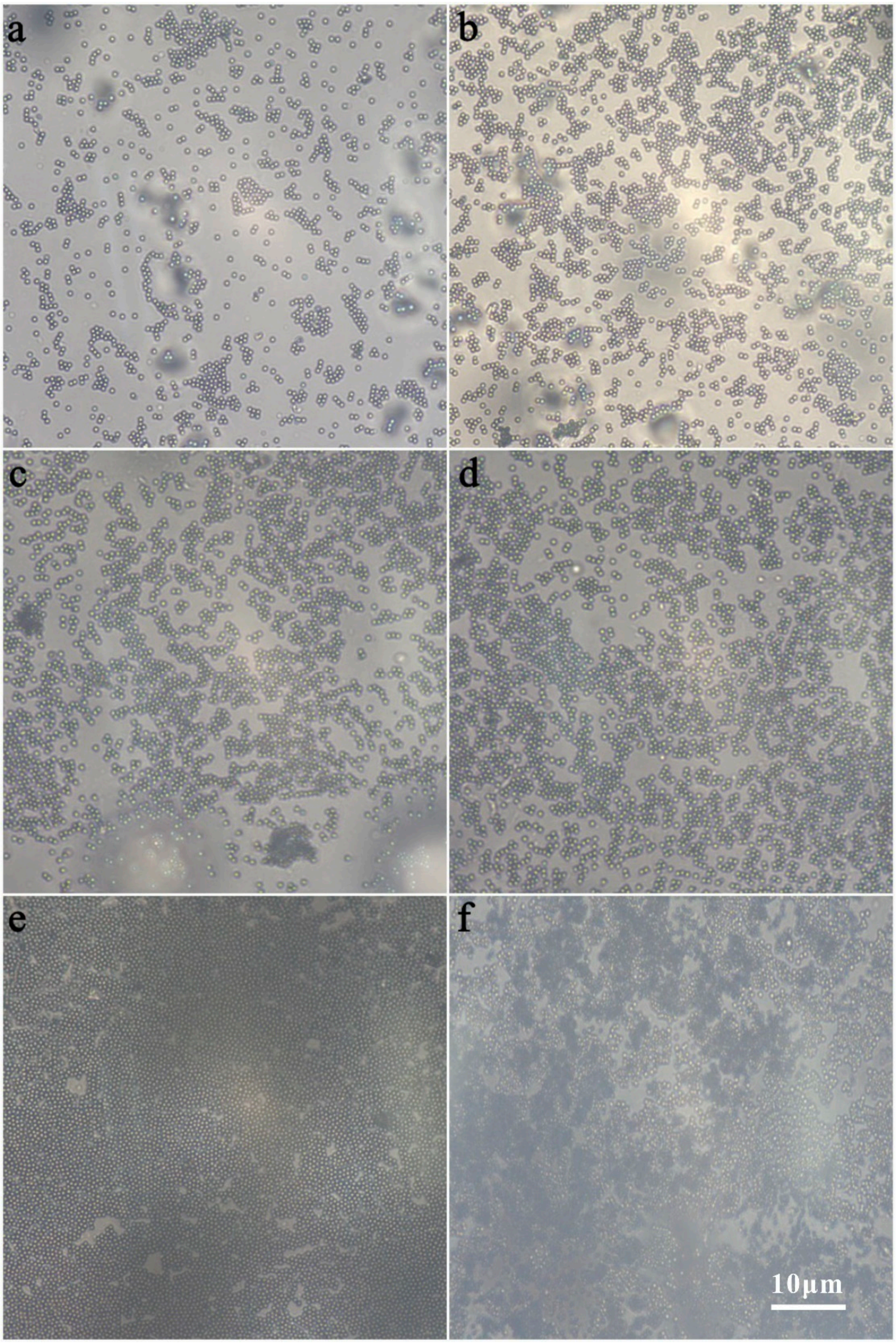

Figure 1. Optical microscope images of PDMS with different concentrations of SMs were assembled on their surface. (×10^7 particles/mL. a: 2.08±0.02; b: 2.76 ± 0.02; c: 3.45 ± 0.02; d: 3.99 ± 0.02; e: 4.35 ± 0.02; f: 5.06 ± 0.02).

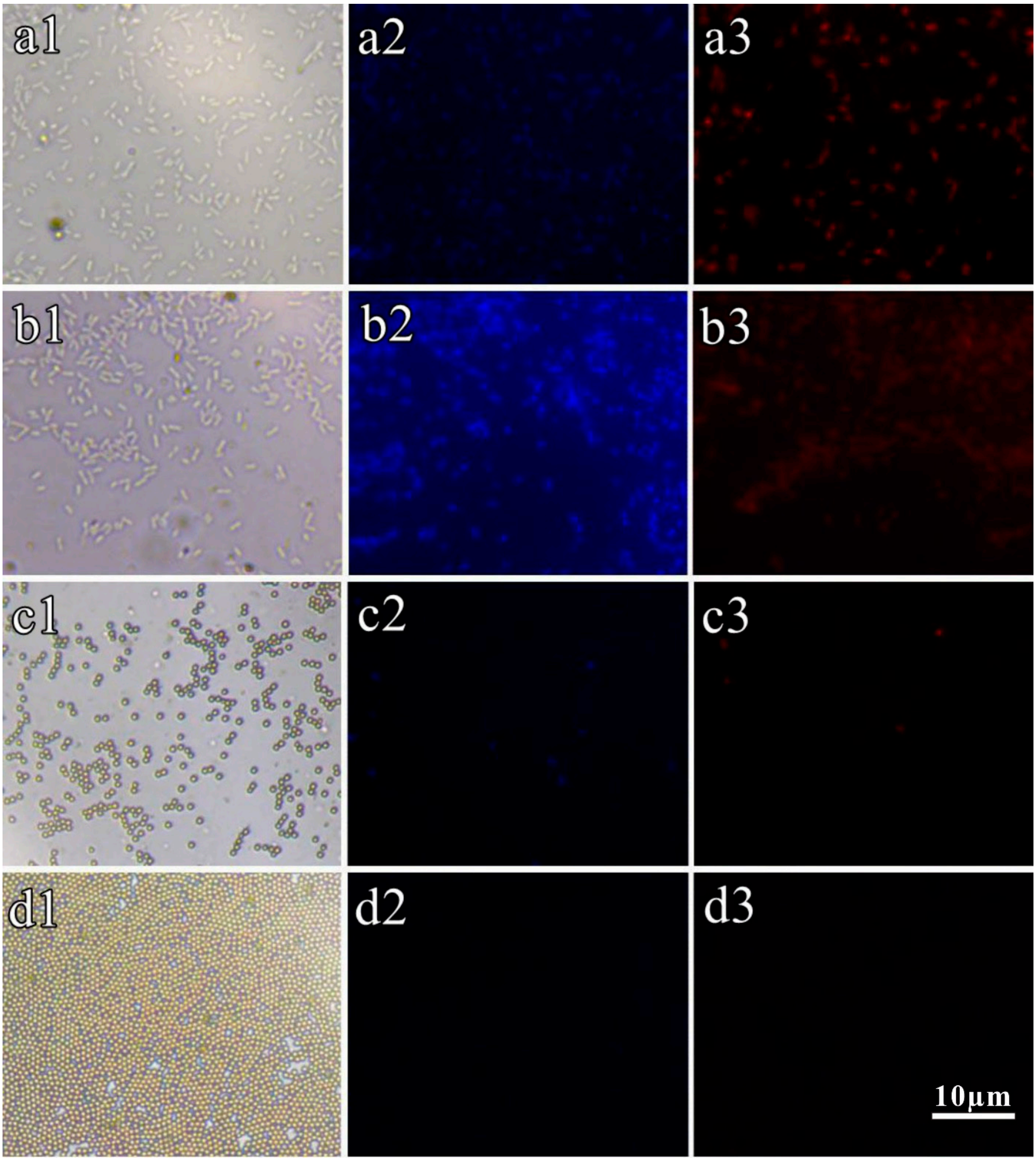

Figure 2. Growth of *E. coli* on the surface of PDMS, PDMS-SMs, PDMS-SMs-AgNPs (sparse/tight). Fluorescent microscopy images of *E. coli* after incubation with (**a**) PDMS; (**b**) PDMS-SMs; (**c**) Sparse AgNPs coated PDMS; (**d**) AgNPs coated PDMS tightly. Cells with blue fluorescence represent all bacteria (2), whereas the red images are representative of dead bacteria (3).

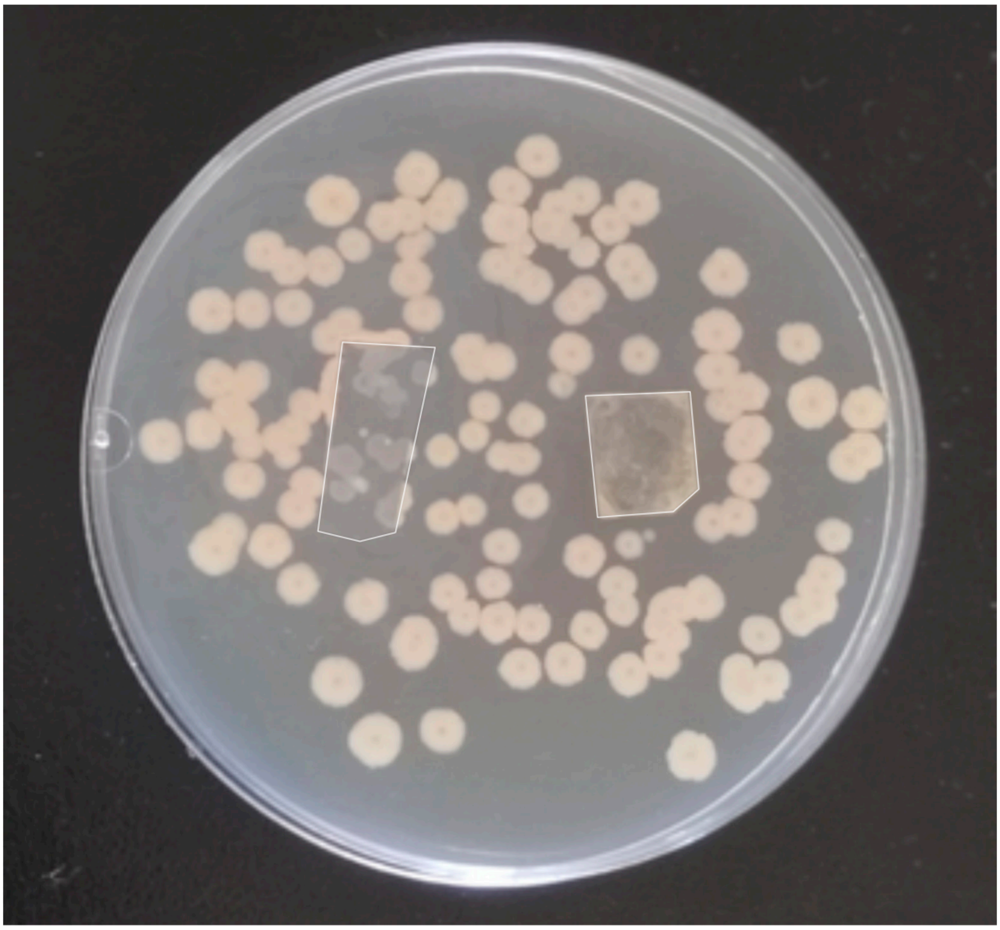

Figure 3. ZOI assay of PDMS (left) and PDMS-SMs-AgNPs (right) with *Bacillus subtilis* after 24 h incubation at 37 °C.

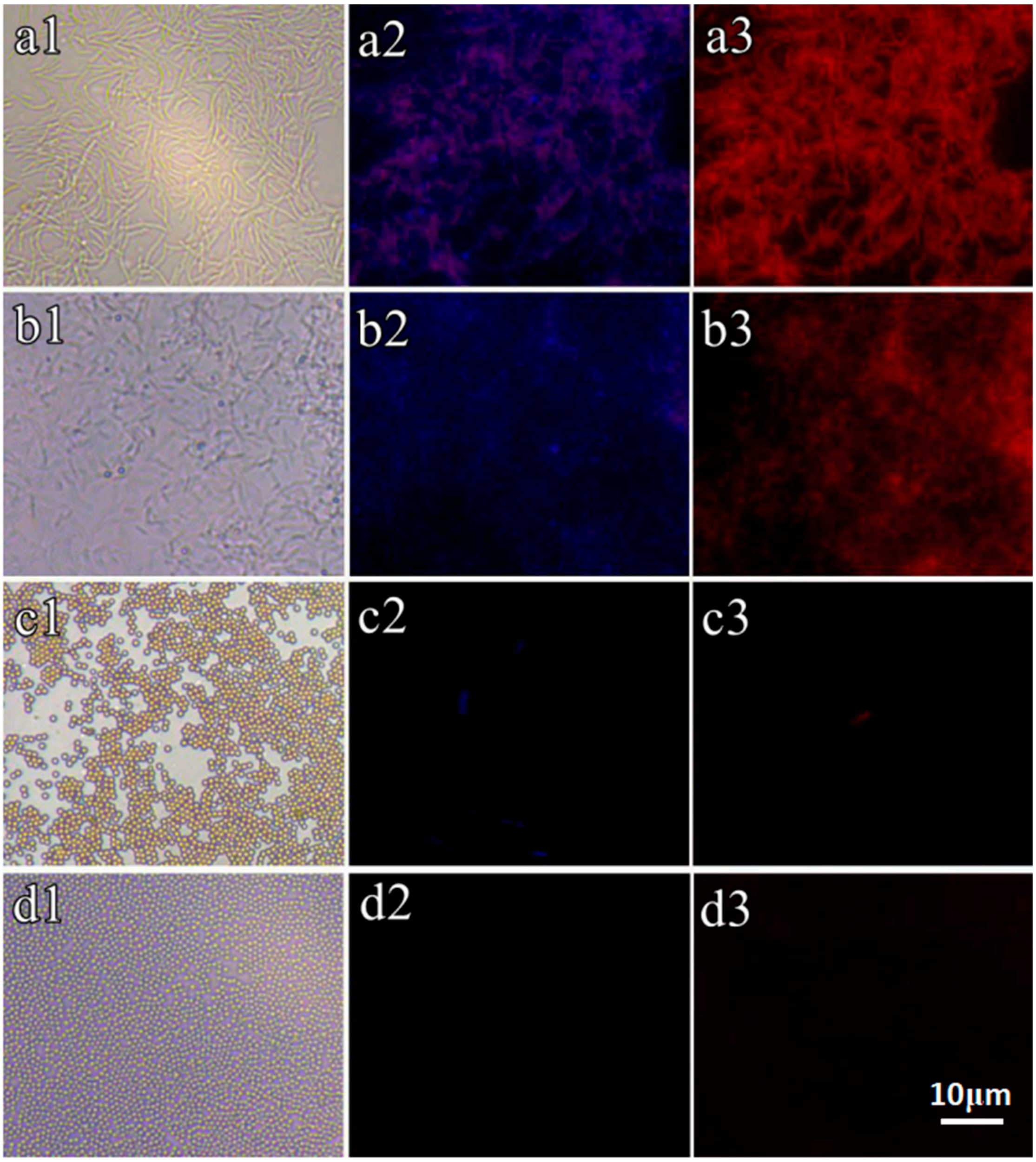

Figure S4. Bright field (1) Fluorescent microscopy (2, 3) images of *Bacillus subtilis* after incubation with (**a**) PDMS; (**b**) PDMS modified with SMs; (**c**) Sparse AgNPs coated PDMS; (**d**) AgNPs coated PDMS tightly. Cells with blue fluorescence represent all bacteria (1), whereas the red images are representative of dead bacteria (3).

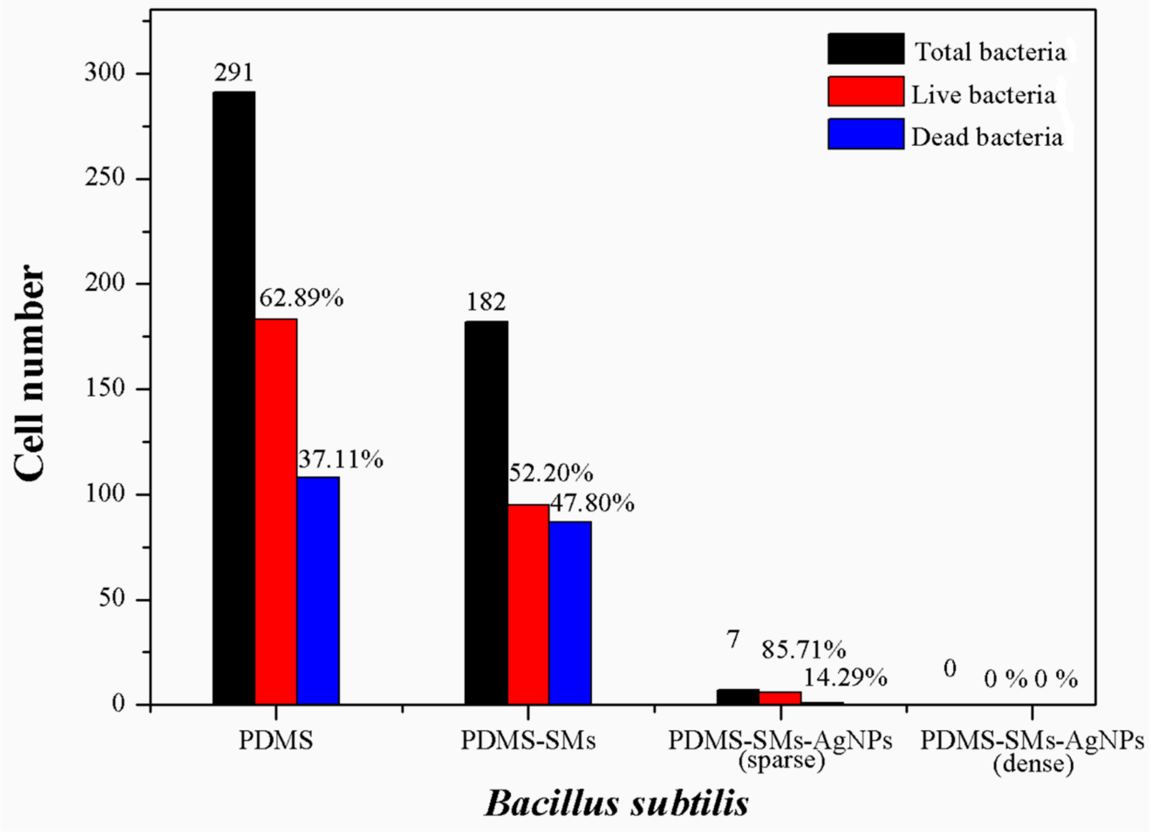

Figure S5. Cell numbers of *Bacillus subtilis* growing on the PDMS, PDMS-SMs, sparse/tight SMs-AgNPs modified PDMS. There is no *Bacillus subtilis* observed on the tight PDMS-SMs-AgNPs.
